# The First Detection of Co-Infection of Double-Stranded RNA Virus 1, 2 and 3 in Iranian Isolates of *Trichomonas vaginalis*

**DOI:** 10.18502/ijpa.v15i3.4200

**Published:** 2020

**Authors:** Farah BOKHARAEI-SALIM, Abdoulreza ESTEGHAMATI, Khadijeh KHANALIHA, Maryam ESGHAEI, Tahereh DONYAVI, Borna SALEMI

**Affiliations:** 1. Department of Virology, School of Medicine, Iran University of Medical Sciences, Tehran, Iran; 2. Research Center of Pediatric Infectious Diseases, Institute of Immunology and Infectious Diseases, Iran University of Medical Sciences, Tehran, Iran; 3. Vice Chancellor for Healthcare, Iran University of Medical Sciences, Tehran, Iran; 4. Student Research Committee, School of Medicine, Iran University of Medical Sciences, Tehran, Iran

**Keywords:** Virus, *Trichomonas vaginalis*, dsRNA virus, Iran

## Abstract

**Background:**

The Totiviridae family includes a number of double-stranded RNA viruses that can infect *Trichomonas vaginalis*. Some *T. vaginalis* isolates are infected with one or more double-stranded RNA (dsRNA) viruses. In this study, different strains of double-stranded RNA virus in Iranian isolates of *T. vaginalis* were evaluated for the first time in Iran.

**Methods:**

Vaginal swabs were collected from 1550 participants who were referred to hospitals associated with Iran University of Medical Sciences, Tehran, Iran from June to November 2018. *T. vaginalis* isolates were cultured in Diamond’s modified medium. After the extraction of nucleic acids using a DNA/RNA extraction kit, RT-PCR was performed and PCR products were purified and sequenced.

**Results:**

In general 9 (0.6%) isolates were confirmed as *T. vaginalis* among 1550 collected vaginal samples. Among 9 isolates of *T. vaginalis*, three of them were infected with TVV1. One isolate has multiple infections with *T. vaginalis* virus (TVV1, TVV2 and TVV3) as coinfection. The nucleotide BLAST indicated that the *T. vaginalis* virus 1(TVV1) isolates were most closely related to TVV1-OC5, TVV1-UR1-1.The *T. vaginalis* virus 2 (TVV2) sequence had also a similarity with TVV2-UR1-1, TVV2-UR1 and TVV2-OC3. The sequence of *T. vaginalis* virus 3(TVV3) had similarity with TVV3-OC5, TVV3-UR1-1 and TVV3-UR1.

**Conclusion:**

Three dsRNA viruses *T. vaginalis* virus (TVV1, TVV2 and TVV3) were detected using RT-PCR in *T. vaginalis* Iranian isolates. The coinfection of TVV1, TVV2 and TVV3 in one isolate of *T.vaginalis* was observed for the first time in Iran.

## Introduction

*Trichomonas vaginalis* is a protozoan parasite that resides in the human genitourinary tract and causes trichomoniasis (1). Patients with trichomoniasis have a wide range of clinical symptoms ranging from vaginitis, cervicitis, urethritis and irritation to asymptomatic presentation (2).

Trichomoniasis are associated with premature delivery, low birth weight and cervical cancer, it also increases the risk of infection by human immunodeficiency virus (HIV) (3–5). The first line treatment for trichomoniasis is metronidazole; however, resistance to metronidazole was observed in some occasions (6). The prevalence rate of trichomoniasis in Iran ranges from 2 to 8% and likely up to 30% among high-risk populations (2). Pathogenesis and virulence factor of *T.vaginalis* is still unknown (1).

These parasites are infected with one or more double-stranded RNA (dsRNA) viruses (TVVs) from genus *Trichomonas* virus and Totiviridae family (7). The virus has been formed in the cytoplasm close to the plasma membrane or near the Golgi complex. Viruses have different sizes (33–200 nm) and shapes (filamentous, spherical, and cylindrical particles) (8).

*Totiviridae* is a family that has 5 genera including *Leishmania* virus, *Giardia* virus, *Totivirus*, *Victoria* virus and *Trichomonas* virus. Different species of *T. vaginalis* virus (TVV) have been identified including TVV1, TVV2, TVV3, and TVV4 according to genome sequences and phylogenetic analysis (1, 9).

Double-stranded RNA virus genome encodes a viral capsid protein (CP) and a viral RNA-dependent RNA polymerase (RdRp) in 2 overlaps of open reading frames (ORFs) (1). The biological significance of *T. vaginalis* infection by TVVs remains poorly understood. It seems that TVV infection is generally a noncytopathic phenomenon that leads to stable and permanent infections. Viruses can be classified as either cytopathic meaning that cells are killed during the infection, or noncytopathic meaning that their replication programs are benign by stopping the process of proapoptotic mechanisms (10). Infections of the protozoan host occurs without an extra-cellular transmission phase (11) and transmission of TVV just happens through cell division and also during mating (9, 12).

*T. vaginalis* infection by TVVs could modulate the pathogenicity of *T. vaginalis* infections by expression of a highly immunogenic *T. vaginalis* protein, P270 (13) and also the expression of cysteine proteinases that are assumed to be virulence factors (14). Genotype of *T. vaginalis* infected with TVV-1 viruses was evaluated by PCR-RFLP in a study (15).

The cDNA sequences of only TVV1 strains in CP region to date have been reported in Iran. Based on these sequences, phylogenetic analysis has been performed according to some new TVV1 sequences in comparison with different TVVs in the family of Totiviridae (16). In this study, different strains of dsRNA virus in Iranian isolates of *T. vaginalis* were evaluated for the first time in Iran.

## Materials and Methods

### Study population

Overall, 1550 vaginal samples were collected from women who were referred to hospitals associated with Iran University of Medical Sciences (IUMS), Tehran, Iran from June to November 2018.

### Ethical considerations

This study was approved by the Ethics Committee of Iran University of Medical Sciences in accordance with Helsinki Declaration and guidelines (IR.IUMS.REC.1397.1383) and all the participants taking part in the study were in agreement with informed consent

### Collection of the specimens

Vaginal swabs were collected from each participant. At first, wet mount samples with sterile phosphate-buffered saline(PBS; pH 7.3 ± 0.1) were prepared and observed using light microscope with 10X and then 40X magnification and then another swab was inoculated into a culture tube at 37°C in Diamond’s TYM medium with 10% heat-inactivated calf serum, 100 U/mL penicillin and 30 mg/mL streptomycin sulphate. Finally *T.vaginalis* culture was harvested in late log phase and centrifuged at 2000 g for 15 min and pellets were stored at −70°C and isolates were confirmed as *T.vaginalis* by PCR (15,16).

### RNA extraction

*T. vaginalis* samples were washed with sterile PBS and centrifuged at 4°C and 5000 g, for 10 min. The RNA was extracted using the Pure Viral Nucleic Acid Kit Roche (Roche Diagnostics GmbH, Mannheim, Germany) according to the manufacturer’s instructions as previously described (16). The reverse-transcription polymerase chain reaction (RTPCR) was performed in order to detect TVV RNA in *T. vaginalis* positive cases.

cDNA synthesis of RNA was performed at 42°C for 30 min in a 20 μL reaction mixture containing 0.5 μg of total RNA, 20 pmol of random hexamer, 4 μL of 5x reverse transcriptase reaction buffer, 125 μmol dNTPs, 104 U Moloney Murine Leukemia Virus reverse transcriptase (Fermentas GmbH, St. Leon-Rot, Germany), 19.2 U RNase inhibitor (Fermentas GmbH) and 1 μL of diethylpyrocarbonate treated water and finally heated at 70°C for 10 min in order to inactivate the reverse transcriptase (16).

### PCR amplification

PCR was performed using specific primers described before (1, 17) in a 25 μL mixture containing the template (3 μL of RT reaction), 2.5 U *Taq* DNA polymerase, 2.5 μL of 1 x PCR buffer, 20 pmol of each primer, 100 μmol dNTPs and 1.5 mmol MgCl_2_.Amplification was performed as follows: initial denaturing for 5 min at 95°C; 40 cycles of 94°C for 30 s, 51°C for 1 min and 72 °C for 40 s; extension at 72°C for 5 min and finally the PCR products were electrophoresed. Expected bands were found for TVV1 at 569 bp, TVV2 at 625 bp, TVV3 at 438 bp, and TVV4 at 514 bp.

### PCR sequencing

A second round of RT-PCR was done for more confirmation and PCR products were purified using the High Pure PCR Product Purification Kit (Roche Diagnostic, Mannheim, Germany) according to the manufacturer’s instructions and were used for direct sequencing using the dye termination method and an ABI 3730xl sequencer. The new sequences of amplified TVV with RT-PCR method was analyzed using genius version 10.1, blasted and compared with those corresponding to different TVV1-TVV3 (dsRNA viruses belonging to the genus *Trichomonas* virus of family *Totiviridae*) taken from the Gen-Bank database. The protein sequences were determined using the program ExPASy (ca.expasy.org/tools/pi_tool)

## Results

Overall, 9 (0.6%) cases were found as *T. vaginalis* by culture method*;* however, *8* (0.51%) *T. vaginalis* were identified in direct method among 1550 collected vaginal samples.

**Fig. 1:**
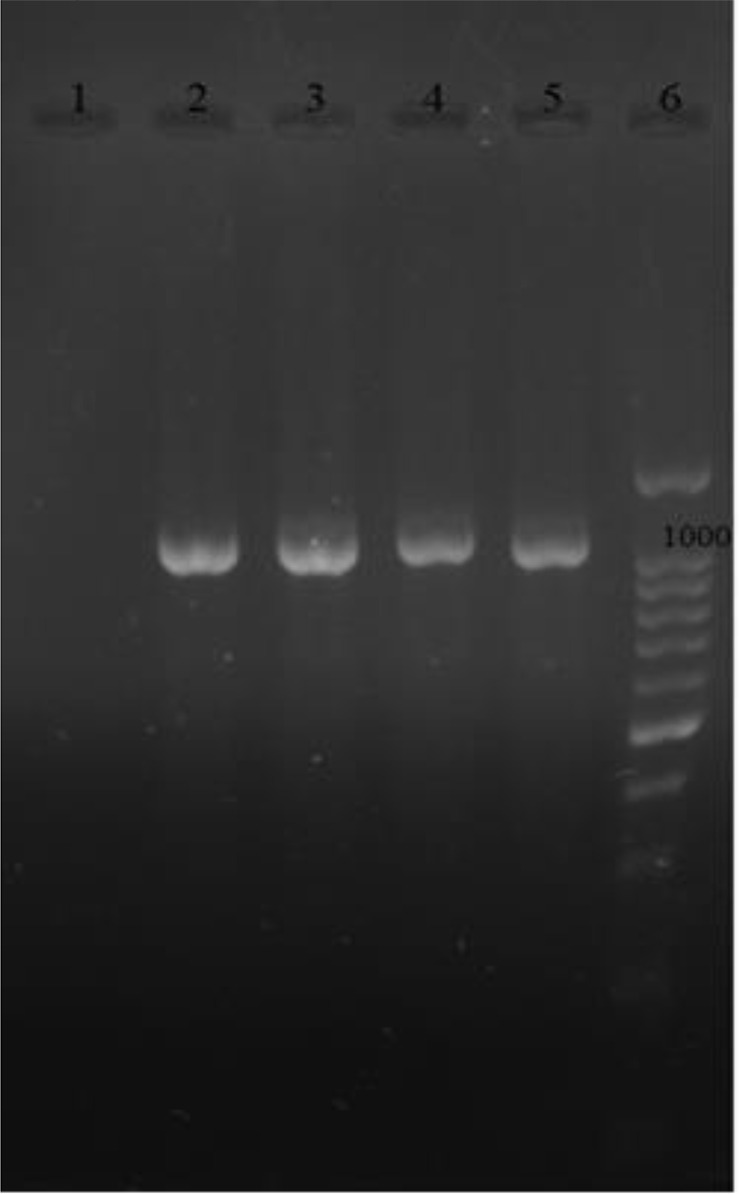
The PCR-amplified products of actin gene of *T. vaginalis* isolates indicated a band of 1100 bp; 1: Negative control; 2: Positive control; 3, 4, 5: Positive *T. vaginalis* isolates; 6: 100bp marker

Among 1550 vaginal samples collected, 9 isolates were confirmed as *T.vaginalis* using actin gene primers. The mean age of the women was 31 ± 4.34 years (ranging from 18 to 45 years). The result of PCR amplification for *T. vaginalis* isolates was evaluated by agarose gel electrophoresis and a band of 1100 bp was observed in (Fig. 1). The *T. vaginalis* isolates were examined by the designed PCR for TVV1, TVV2 and TVV3 and TVV4. In this study, among 9 *T.vaginalis* isolates, three of them were infected with TVV1; one of them was co-infected with TVV1, TVV2 and TVV3. Most prevalent strains were TVV1 (3) followed by TVV2 (1) and TVV3 (1).

**Fig. 2:**
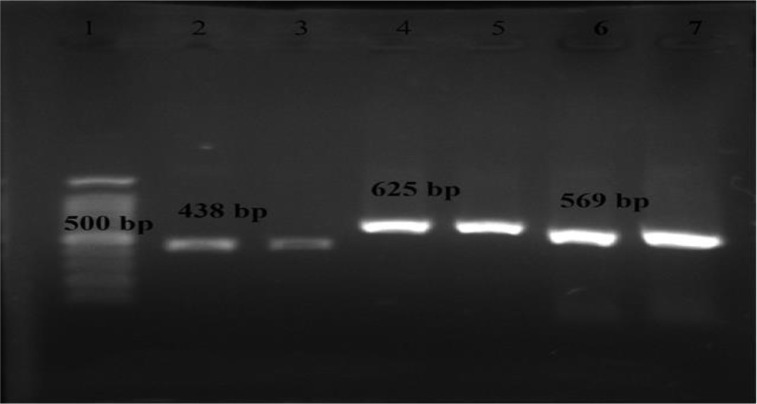
RT-PCR-amplified products of TVVs in Iranian isolates of *T.vaginalis* lane 1: DNA marker (100 bp); lane 2, 3 positive bands of TVV3 at 438bp, lane 4, 5: positive bands of TVV2 at 625 bp, lane 6 and 7 TVV1 at 569 bp

The PCR amplification of different *T. vaginalis* on gel agarose electrophoreses was observed in Fig. 2. The bands of 569 bp for TVV1, 625 bp for TVV2 and 438 bp for TVV3 were found on gel agarose electrophorese (Fig. 2).

The new sequences were submitted in Gen-Bank as MH722257, MH722258, MH722259 (TVV1), MH743216 (TVV2) and MH722260 (TVV3) accession numbers. The result of PCR sequencing confirmed these sequences as TVV1, TVV2 and TVV3.The sequences of TVV1, in this study, showed 92% identity with TVV1-OC5, and 90% with TVV1-URI-1 and TVV1-UR1. The obtained sequences TVV2 had 93% similarity with TVV2-UR1-1, TVV2-UR1 and TVV2-OC3. The sequence of TVV3 had 92% similarity with TVV3-OC5, and 90% with TVV3-UR1-1 and TVV3-UR1.

BLAST TVV1 amino acids showed 96% identity withTVV1, accession number (AED99820.1). BLAST TVV2 amino acids showed 97% identity with TVV2, accession numbers (AKE98370.1 and AED99806.1) and TVV3 had 98% identity with TVV3, accession number AKE98371.1.

## Discussion

Parasitic protozoa, *T. vaginalis* can be infected by one or more subtypes of dsRNA viruses (1). TVV is transmitted vertically during the *T. vaginalis* binary fission (5); however it is lost through long term passages (18).

In this study, we identified three TVVs including TVV1, TVV2, and TVV3 detected from Iranian isolates of *T. vaginali*s using genome sequencing and molecular analysis with other sequences of different TVVs. Furthermore, *T. vaginalis* was detected by PCR in 9 of 1550 women attending hospitals associated with Iran University of Medical Sciences, Tehran, Iran. In general, 33.3% of *T. vaginalis* isolates were infected with dsRNA viruses. Among 9 *T. vaginalis* isolates, three of them were infected with TVV1 that one of them was infected with TVV1, TVV2 and TVV3 at the same time. So, there was one isolate harboring three TVVs, including TVV1, TVV2 and TVV3. Direct sequencing and nucleotide and amino acids BLAST search confirmed the identity of these dsRNA viruses using RT-PCR.

In the previous study, infection to just TVV1 in *T.vaginalis* isolates has been reported in Iran. Phylogenetic analysis confirmed that the Iranian isolates were related to TVV1-OC5, TVV1-UR1 (16). The presence of multiple TVVs in a single isolate of *T.vaginalis* has been reported by some studies (1, 19, 20). The multiple viral infections in a single host has also been reported in the genera *Totivirus* and *Victorivirus* (1). The presence of several TVVs in a single culture may be due to the presence of multiple parasites that were infected with different TVVs or because of synchronous infection of TVVs in a single parasite (21).

Moreover, 44 % *T. vaginalis* isolates were infected with dsRNA virus from different geographic origins (22). The frequency of TVV infection among *T.vaginalis* isolates has been reported as 55% in Cuba (23) and 50% in USA (24). The TVV infection rate of 81.9% was reported in *T. vaginalis* isolates from South Africa (7).

In contrast, two studies conducted in Korea and Tehran reported that only 13.6% (25) and 17.39% of the *T.vaginalis* isolates were infected with TVVs respectively; however only TVV1 was identified in *T. vaginalis* isolates from Iran (26).

Goodman et al conducted a study on *T. vaginalis* isolates and reported that TVV-1 was the most prevalent (77.5 %) of them, followed by TVV-2 and TVV-3 (30%). The less frequent TVVs were related to TVV-4 (10 %). The result of the study also demonstrated that one isolate of *T. vaginalis* could be infected by several TVV species (1). Among three *T. vaginalis* isolates, TVV-2 and TVV-1 were identified and TVV species were associated with two TVV2 and one TVV1 (27).

Rivera et al reported different subtypes of TVV in *T. vaginalis* isolates. In this study among 18 *T. vaginalis* isolates, 35 dsRNA viruses were detected. Various TVV were identified in six of the 18 *T. vaginalis* samples. Phylogenetic analyses in this study proposed monophyly in TVV1 and TVV2, but TVV3 and TVV4 appear paraphyletic (21).

In a study, 8 out of 40 (20 %) *T. vaginalis* isolates were infected with dsRNA viruses. TVV2 was the most prevalent species that was identified in 5 out of 8 (62.5%), followed by TVV4 in 3 out of 8 (37.5%). All isolates infected with TVV were detected from the symptomatic patients. A significant association between the presence of dsRNA viral infection of *T. vaginalis* isolates and symptoms like vaginal discharge, erythema and dysuria was found (*P*<0.01) (17).

Although the result of some studies showed there was no relation between the ages of patients and the viral infection (17, 26), a significant relation between age and TVV infection has been found in the older age of individuals in a study (28).

In the present study, there was no relation between the ages of patients and the viral infection. Although symptoms like vaginal discharge and erythema were found in women infected with TVVs, there was no significant association between the presence of dsRNA viral infection of *T. vaginalis* isolates and symptoms.

The presence of TVV has also been associated the expression of *T. vaginalis* virulence factors. Although this infection makes upregulation of a highly immunogenic protein, P270 (13), the expression of surface P270 was just noticeable in *T. vaginalis* cells infected with TVV2 and TVV3, suggesting that the transcription of the P270 gene is likely regulated by viral factors common in type 2 and type 3 viruses (29).

In the current study, infection with all of the three TVV1, TVV2 and TVV3 species of *Trichomonas* virus were identified in Iran and TVV2 and TVV3 species of *Trichomonas* virus were reported for the first time. BLAST comparisons of the partial nucleotide sequences and amino acids confirmed the viruses as *T. vaginalis* virus.

## Conclusion

This research is the first report of the *T. vaginalis* infection by TVV2 and TVV3 in Iran. The prevalence of TVV subtypes is noticeable in *T. vaginalis* in Iran and three dsRNA viruses TVV1-TVV2 and TVV3 were detected using RT-PCR in *T. vaginalis* isolates. Single and multiple infections to different subtypes of TVV in *T. vaginalis* isolates were observed in this study.
